# An integrated workflow for 2D and 3D posture analysis during vestibular system testing in mice

**DOI:** 10.3389/fneur.2023.1281790

**Published:** 2023-12-01

**Authors:** Yong Wan, Michaela A. Edmond, Colin Kitz, Joseph Southern, Holly A. Holman

**Affiliations:** ^1^Scientific Computing and Imaging Institute, University of Utah, Salt Lake City, UT, United States; ^2^Department of Neurology, University of Utah, Salt Lake City, UT, United States; ^3^Department of Biomedical Engineering, University of Utah, Salt Lake City, UT, United States

**Keywords:** balance, inner ear, coordination, multisensory, spatial orientation, AI, ML

## Abstract

**Introduction:**

Posture extraction from videos is fundamental to many real-world applications, including health screenings. In this study, we extend the utility and specificity of a well-established protocol, the balance beam, for examining balance and active motor coordination in adult mice of both sexes.

**Objectives:**

The primary objective of this study is to design a workflow for analyzing the postures of mice walking on a balance beam.

**Methods:**

We developed new tools and scripts based on the FluoRender architecture, which can interact with DeepLabCut (DLC) through Python code. Notably, twenty input videos were divided into four feature point groups (head, body, tail, and feet), based on camera positions relative to the balance beam (left and right), and viewing angles (90° and 45° from the beam). We determined key feature points on the mouse to track posture in a still video frame. We extracted a standard walk cycle (SWC) by focusing on foot movements, which were computed by a weighted average of the extracted walk cycles. The correlation of each walk cycle to the SWC was used as the weight.

**Results:**

We learned that positions of the camera angles significantly improved the performance of 2D pose estimation (90°) and 3D (45°). Comparing the SWCs from age-matched mice, we found a consistent pattern of supporting feet on the beam. Two feet were consistently on the beam followed by three feet and another three feet in a 2-3-3 pattern. However, this pattern can be mirrored among individual subjects. A subtle phase shift of foot movement was also observed from the SWCs. Furthermore, we compared the SWCs with speed values to reveal anomalies in mouse walk postures. Some anomalies can be explained as the start or finish of the traversal, while others may be correlated to the distractions of the test environment, which will need further investigation.

**Conclusion:**

Our posture analysis workflow improves the classical behavioral testing and analysis, allowing the detection of subtle, but significant differences in vestibular function and motor coordination.

## 1 Introduction

The vestibular system functions by detecting head position, movement, and relative gravity in space through a multisensory integration of vestibular input, eye movement, posture, orthostasis, and proprioception [Angelaki and Cullen (1), Review]. Testing the vestibular system, either passively or actively, in rodents has presented many challenges. Two well-established protocols are walking on a rotarod and balance beam, which have been used to indirectly measure active vestibular function. Video recordings of rodents performing behavioral tests provide additional measurements (2). Further assessment of mouse movements has been developed with markerless algorithms such as DeepLabCut (DLC) that provide quantitative tracking of limbs and joints (3). The analysis of such videos demands the digitization of mouse postures as joint locations from each video frame. These joints are also called landmarks or feature points and are represented by their 2D coordinate values in an image. Generating the posture information accurately can be labor-intensive work, as each video commonly contains several hundred frames and each frame several tens of feature points. The rise of machine learning (ML) and artificial intelligence (AI) provides a feasible path to acquire posture information from videos in large quantities. Several neural network-based methods for pose estimation have been developed for markerless detection of anatomical feature points [i.e., landmarks or keypoints (4–7)]. An open-source method called OpenPose enables real-time key body keypoints to be tracked from a multi-person human detection library (5, 8), and has been used as part of a 3D markerless system to calculate joint angles during a normal gait (9). Human pose estimation is the computer vision task of localizing joints in an image or video (e.g., shoulder, elbow, wrist, ankle, foot, face). One implementation, DeepLabCut (DLC), originally designed for tracking animal behavior (7), has subsequently utilized a markerless neural-network approaches in human movement studies (10).

However, there are limitations of off-the-shelf pose-estimation packages when being applied in practice. First, training examples need to be manually generated. An easy-to-use and efficient tool is still needed for manually tracking feature points from video frames for AI-based training to properly start. The quality of training examples has significant influence on the ML outcome. The tool for manual tracking also needs to generate accurate examples. Second, pose estimation provides positions of body feature points for further posture analysis to extract information about movements and behaviors. Inaccuracies in AI-based pose estimation results introduce significant biases to subsequent posture analysis and need to be reduced by user guidance. The accuracy requirements of pose estimation are less strict than those of posture analysis. Common applications of pose estimation include gaming, robotics and animation, and similar algorithms. For these applications, it is usually sufficient to predict the location of a body feature points to within 5 to 10 cm. When calculating joint angles for posture analysis, this magnitude of error is unacceptable [Seethapathi et al. (11), Review]. For example, a foot slip has subtle speed changes that can be masked by an inaccurate tracking of several pixels. Thus, pose estimation algorithms cannot simply be used out of the box. A tool to correct errors introduced by AI is needed to guarantee the validity of posture analysis. Finally, the speed values derived from pose estimation lack the descriptive information to quantitatively evaluate and compare movements. Patterns of movements need to be further detected and extracted from speed values, which itself is an application of ML for posture analysis.

**Figure 1:**
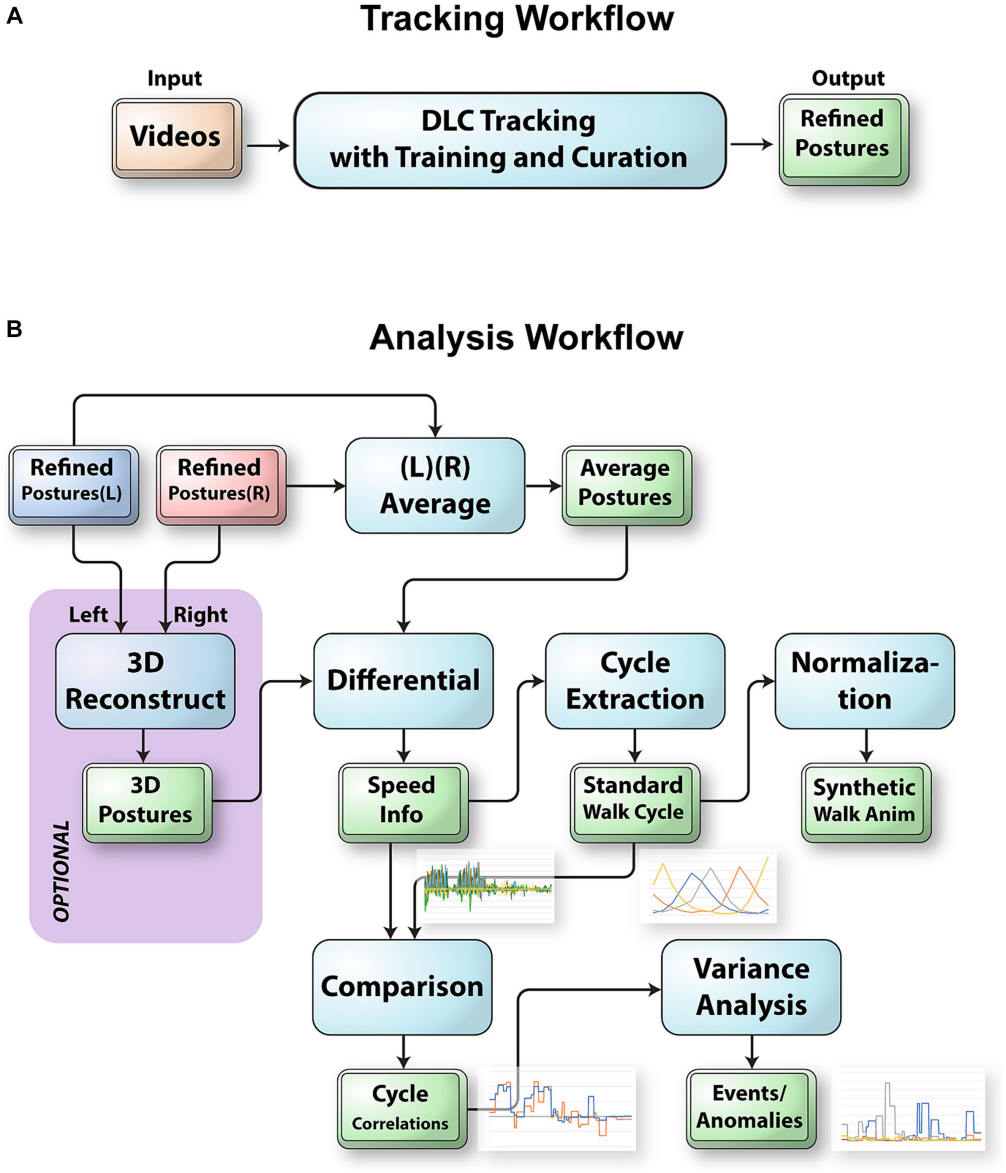
The workflow to track and analyze videos of mice traversing the balance beam. **(A)** Integration of DLC into FluoRender to track raw videos. FluoRender’s ruler tools were used to generate example postures on select video frames. These examples were saved into a DLC project for training a DLC model. Crude postures were generated by applying the model to full videos. The ruler editing tools of FluoRender were used to curate the crude postures and achieve precise tracking results. **(B)** The left and right postures were merged depending on the viewing angles of the video camera setups. For the 45° videos, we reconstructed 3D postures using a computer vision workflow with autocalibration. For the 90° videos, we computed the average postures from left and right postures. Then, we computed the speed values from the postures. We extracted every walk cycle from the speed data and computed a weighted average as the standard walk cycle. The SWC was first used to generate synthetic walk animations. We also compared the SWC with the speed data for each video to detect special events and anomalies in the videos.

Here, we present an integrated workflow and accompanying tool for posture analysis that combines visualization, manual tracking, AI-based pose estimation, and pattern extraction (Figure 1). Our main platform is FluoRender, which supports viewing time-dependent images and tracking feature points. We added a Python interpreter into the FluoRender system that allows the integration of DLC functions for pose estimation. We improved the ruler functions in FluoRender to provide efficient and intuitive manual tracking capabilities. Furthermore, we developed a series of scripts based on the FluoRender architecture for extracting patterns as the standard walk cycle (SWC) of a mouse on a balance beam, so that most tasks in our workflow were automated by script executions. The extraction of a standard pattern allows users to compare libraries and detect anomalies in gait analysis in the mouse. Our new posture analysis workflow extends established markerless tracking methods by providing new measurements beyond the position and speed information to quantitatively describe the movements and behaviors of the mouse traversing a beam. Moreover, it is also a versatile tool for a variety of behavior-related balance and motor coordination applications including Parkinson’s disease, cerebellar diseases, acoustic neuromas, and traumatic brain injuries.

## 2 Methods

### 2.1 Animals

All animal experiments carried out in this study were conducted at the University of Utah and approved by the University of Utah Institutional Animal Care and Use Committee in accordance with NIH guidelines.

Both male and female C57BL/6 J (C57BL/6 J jax.org/strain/0000664), a dual Cre-dependent reporter Polr2a-based GCaMP5G-IRES8 tdTomato, referred to as PC::G5-tdT (12), and a Gad2-IRES-Cre knock-in mouse driver line, referred to as Gad2::Cre (13), were used in these experiments. Both transgenic breeding pairs were obtained from The Jackson Laboratory (Polr2atm1(CAG-GCaMP5g, tdTomato)Tvrd jax.org/strain/024477; Gad2tm2(cre)Zjh jax.org/strain/010802). The parental strain C57BL/6 J carries a cadherin 23 mutation leading to early onset hearing loss. However, age-related vestibular dysfunction is considered minimal in these mice, with no evidence of early-onset vestibular dysfunction (14). First-generation heterozygous transgenic offspring were used in all experiments. Mice were genotyped using real-time PCR [probes: “Polr2a-3,” “GCamp3-1 Tg,” and “tdRFP”; (Transnetyx, Inc.)]. Mice were housed in a 12 h light/dark cycle; light started at 6 a.m. Mice had unlimited access to water and Envigo 2,920X food. Temperature for housing and vestibular testing suites were monitored and maintained between 20 and 23°C.

### 2.2 Balance beam

**Figure 2:**
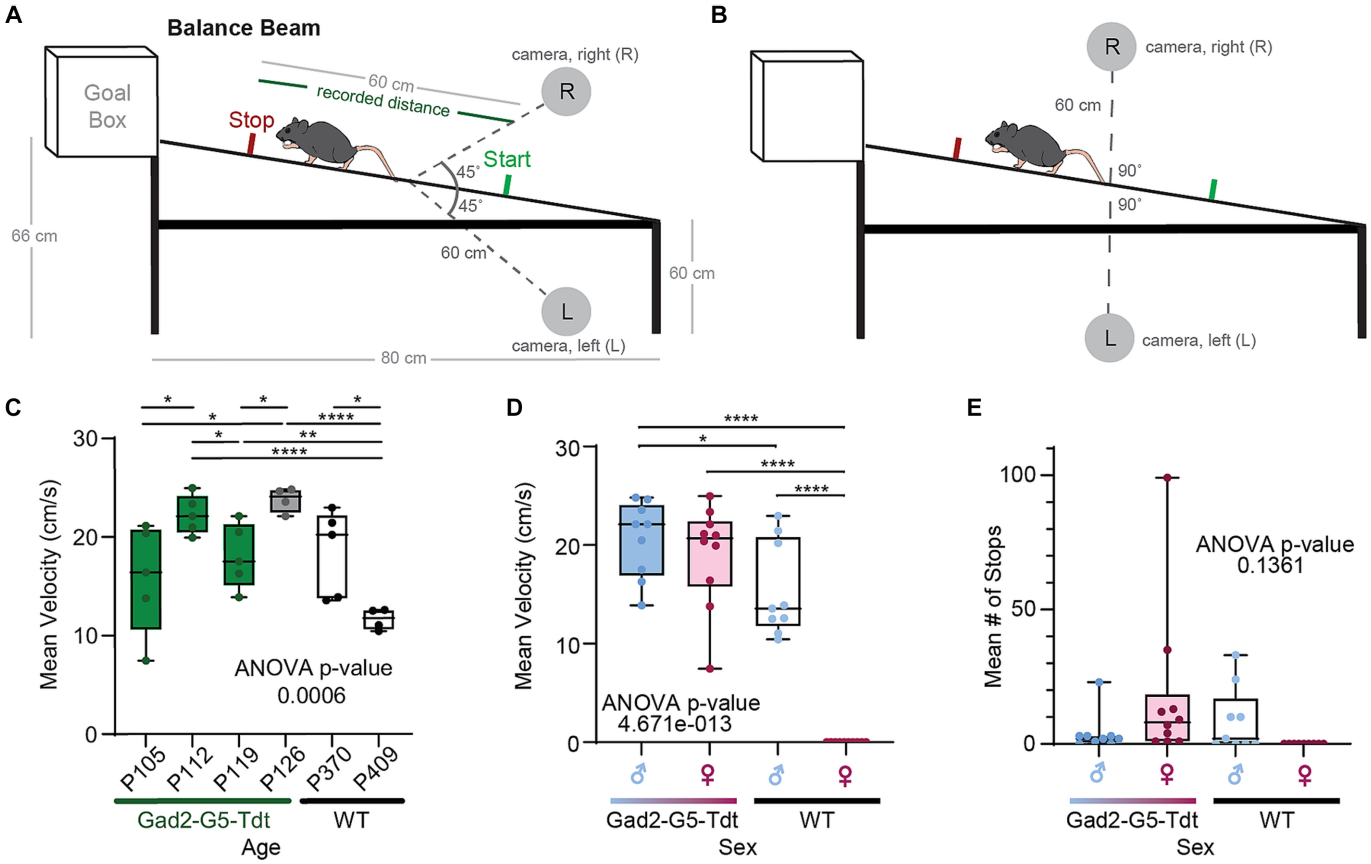
Balance beam testing across different adults ages, sexes, and strains. **(A)** An illustration of balance beam (mouse not drawn to scale) with two cameras pointing to the center of the beam at 45° angles. **(B)** An illustration of the beam with two cameras facing each other on two sides of the beam. **(C)** Mean velocity (cm/second) for each adult mouse to traverse the beam between 4 to 5 trials (ages (P)ostnatal day 105 to P409). **(D)** Summary of mean velocities for males and females in Gad2-G5-tdT and wild type (WT) mice. **(E)** Summary of mean number of stops along the balance beam for males and females in Gad2-G5-tdT and wild type (WT) mice.

The vestibular function of adult mice from both sexes was tested using a balance beam apparatus in this study. A custom balance beam and scaffold were built (Figures 2A,B), adapted from Carter et al. (15) and Tung et al. (16). The stainless-steel beam has a diameter of 2.54 cm and was stationary in this study. The start of the balance beam was positioned 60 cm above the ground, and the end of the beam (i.e., goal box) was positioned 66 cm above the ground. Recordings were made when mice traversed 60 cm in the middle of the beam, which was demarcated with tape. Mice were given four practice trials with sequentially longer distances to walk along the beam, with the goal of achieving one successful full-length distance of 60 cm section before trial recordings. Following each trial, the mouse was placed back in his/her housing with food and water, to rest for a minimum of 2 min. All trials were video recorded at 30 frames per second (fps) for subsequent analysis.

### 2.3 Balance beam data acquisition

Each mouse walked from the start of the beam into a goal box. The process of walking across the middle 60 cm was filmed by two cameras positioned at two angles relative to the beam. Videos were recorded with smartphone cameras (iPhone7) with a 2.4 aperture, 12 MP telephoto lens, and 30 fps. Each of camera was secured to a tripod located 60 cm away from the balance beam on either side. For the first configuration were positioned at the start of the beam and pointed to the center of the beam at 45° angles (A), and in the second configuration the two cameras faced each other on either side of the balance beam [left (L) or right (R)], pointing to the center of the beam at a 90° angle (B). Cameras were placed so that the entire beam was visible. All videos were taken with camera settings of 30 fps and high definition (HD). The test was repeated 5 times for each mouse.

### 2.4 Feature point creation and editing

**Figure 3:**
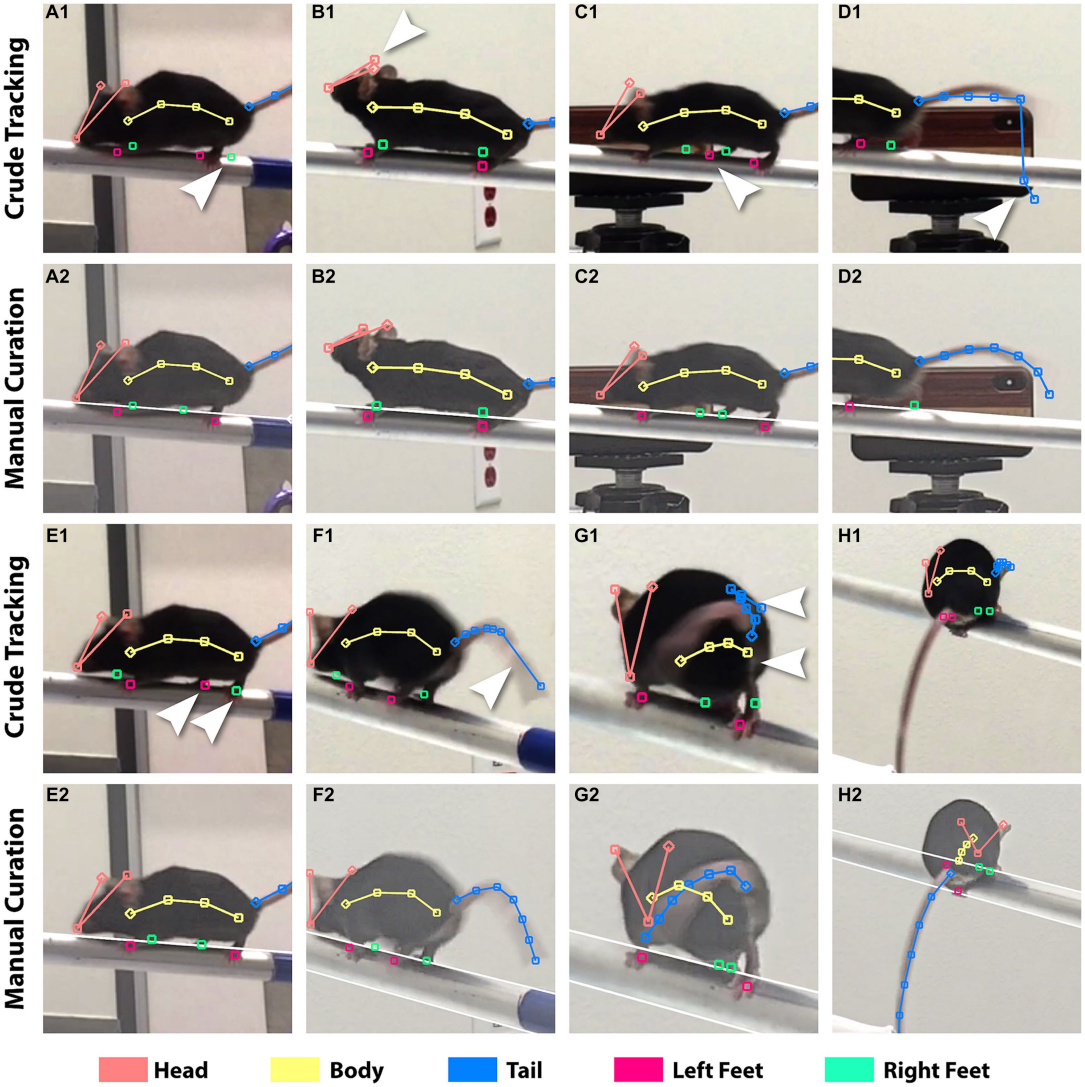
Incorrect tracking from DLC versus manual correction. The crude tracking rows show results from DLC. The manual curation rows show the results after fixing. The brightness of the videos was adjusted in FluoRender to aid feature discerning. Arrows point to the incorrect feature points. **(A1,2)** A blurry rear right foot caused incorrect tracking for both rear feet. **(B1,2)** Left and right ear tips were not correctly identified. **(C1,2)** Background object confused the tracking. **(D1,2)** Tracking of the tail failed because of the influence from the beam. **(E1,2)** Left and right rear feet switched positions. **(F1,2)** The feature points for the tail were not evenly distributed. **(G1,2)** Incorrect identification of the tail and body from self-occlusion. **(H1,2)** DLC was unable to track this atypical posture because it was not provided for training.

We determined key feature points on the mouse to track its posture in a still video frame. We used the ruler tools in FluoRender for the placement of feature points. We determined an 18-point configuration to be sufficient for tracking postures while maintaining work efficiency. The points were organized into 4 groups: 3 points for the head consisting of the left auricle (ear tip), the right auricle, and the nose tip, 4 for the torso (body), 7 for the tail, and 4 for the feet (left front and back, right front and back). Two different types of rulers in FluoRender were used to keep track of the grouping of feature points. For the head, a polyline of connected points was created by clicking on the ear and nose tips. For the body and tail, a polyline was created by the pencil tool in FluoRender, which allowed drawing by a freehand stroke. Each foot was labeled by a single point. We colored the coded points by groups whereas foot points were colored distinctively for ease of identifying the left from right foot (Figure 3). Feature points were only created for an initial video frame, where a mouse was fully visible. Feature point count and grouping were maintained over time. We only modified point positions to track mouse postures in subsequent video frames. We developed three tools for FluoRender to edit feature points efficiently. The “move tool” moves one point at a time by allowing the user to click on the point and drag it around. The “magnet tool” avoids dragging by attracting the closest point after the user clicks on the desired location. To edit multiple points on a polyline ruler together, the magnet tool also lets the user draw a freehand stroke and attracts the points from the closest ruler to the stroke. To allow fine-tuning of the length of a ruler under editing, we designed a “redraw tool” that works similarly to the magnet tool but controls ruler length using the freehand stroke. In contrast, the magnet tool maintains the distance between two points when they are repositioned. The design of these editing tools is aimed at optimizing efficiency for full manual tracking of entire videos. Nevertheless, our tools also expedited generating deep-learning examples and curating deep-learning results when the DLC library was integrated with FluoRender.

### 2.5 DeepLabCut training and tracking

We installed and configured the DeepLabCut (DLC) library on the same computer running FluoRender. We added a Python interpreter into FluoRender, which can function with DLC (17). We used FluoRender to generate training examples. The 20 input videos were divided into 4 groups based on camera positions (left and right) and viewing angles (45° and 90° from the balance beam; Figures 2A,B). Each group was trained and tracked with a dedicated DLC model (7). We selected video frames covering typical postures and various locations on the beam from each video group and generated feature points using the FluoRender rulers. For each model, the frame number required for training varied from 23 to 31. We ensured that the 18-point configuration was consistently maintained for all models. In fact, the feature point configuration was only created once at the first selected frame for each model. All subsequent example frames were tracked by the editing tools we developed for FluoRender, such as the magnet and redraw tools. To further automate DLC-based training and tracking, we developed two FluoRender scripts (video_train and video_analysis, included with FluoRender releases on github.com). Once the example frames were generated, we executed the first FluoRender script that performed a series of operations. It exported the example frames with rulers to a DLC project, launched the DLC within the Python environment, and started model training using the project. The script contained a default iteration count of 300 K, which we found to be a reasonable trade-off between model accuracy and time cost. Training was computed on an nVidia Quadro M6000 graphics card with GPU acceleration configured for DLC. The time cost of training is proportional to video resolutions. It took about 12 h to train a model for videos at 1280 × 720 resolution and about 24 h for 1920 × 1080 resolution videos. Once a model was trained, we tracked all videos in its group using a second FluoRender script (18), which again called the Python code and leveraged the DLC project. The script read back the tracking results from DLC and converted them to FluoRender rulers for instant examination using FluoRender’s visualization. The time cost for tracking was negligible, as tracking results for a full video were generated when the video was viewed.

### 2.6 Tracking curation

The tracking from DLC contained erroneous and imprecise placement of feature points. The quality of tracking results is limited by video clarity, which is affected by a multitude of factors including image sensor size, optical resolution, and viewing angle. Hence, simply increasing the iterations for training did not make a significant improvement. On the other hand, improving the model by iteratively increasing the training examples is a trial-and-error process, which would significantly increase the time cost as each training session took about 1 day. Given the amount of data and computing hardware available, we struck a balance between manual and machine time to ensure high tracking precision. We manually curated the crude tracking by designing a streamlined curation workflow and leveraging FluoRender’s ruler editing functions, which were made for manual work productivity. In FluoRender, we carefully examined every feature point in every frame. We also took advantage of FluoRender’s image enhancement settings to adjust the brightness and contrast so that features were easily discerned. Any erroneous or imprecise tracking was corrected by FluoRender’s editing tools, similar to how the training examples were created. Figure 3 lists typical issues from crude DLC tracking compared to manually curated results. The curation work was timed for an evaluation of efficiency. A total of 4,292 frames were examined or corrected using 985 min (16.42 h). The average speed of curation was 4.36 frames/min, or 13.76 s/frame. The average speed for each video ranged from 2.44 frames/min to 8.97 frames/min because work intensity varied for issues at different complexity levels.

### 2.7 3D reconstruction/left–right averaging

**Figure 4:**
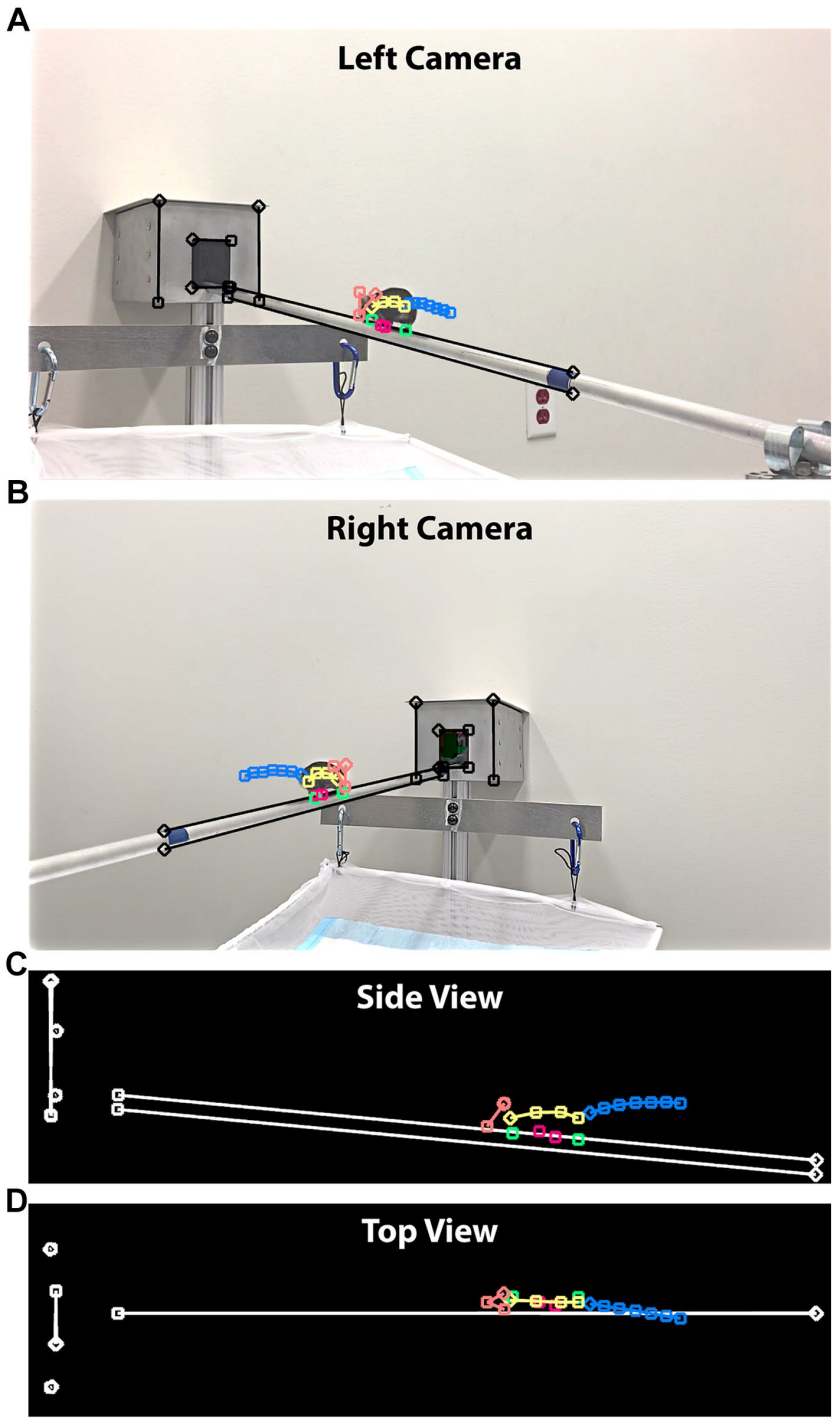
3D posture reconstruction with cameras positioned at 45°. **(A)** A still frame from the left-side camera. In addition to the feature points tracking the mouse movements, we drew straight lines in three groups, tracing the beam and edges of the box. **(B)** A still frame from the right-side camera. The same straight lines were drawn to establish a 3D world coordinate system. **(C)** The side view of the reconstruction result. Both the beam length and inclination angle were provided to map the reconstructed lengths to their physical values. **(D)** The top view of the reconstruction result.

We adopted a dual camera configuration to improve tracking accuracy, as all 18 feature points were not always clearly visible from just one camera. Both advantages and disadvantages were present between the two viewing angles of 90° and 45°. A significant advantage of adopting the 45° camera viewing angle was the ability to reconstruct mouse postures three-dimensionally. DLC included functionality for 3D reconstruction from a stereo camera configuration (7). However, DLC’s mandatory camera calibration limited its use in practice because of the lack of awareness for calibration and the fact that a calibrated setup could be accidentally changed by operators. We developed a script in FluoRender to perform a 3D reconstruction of feature points independent of DLC. A reconstruction workflow described by Hartley and Zisserman (19) was implemented in steps. It calibrated the cameras using only information from the videos, which was input by the user tracing 3 pairs of corresponding parallel lines from two camera views (Figure 4). We traced the upper and lower edges of the balance beam, the outer edges of the destination goal box, and the edges of the goal box entrance. Given these lines, we first computed the fundamental matrix from their endpoint coordinates using the least median squares (LMS) algorithm. We fixed the left camera matrix to canonical and derived the right camera matrix from the fundamental matrix using a QR decomposition. Then, we updated the camera matrices to enforce perpendicularity and parallelism among the line pairs in 3D. The coordinate system of the reconstructed scene was transformed, so that the line pairs were aligned with the XYZ axes: beam to X, box edges to Y, and entrance edges to Z. Finally, the values from physical measurements (beam length and inclination angle) were used to perform a shear correction and then map all point coordinates to their physical scale.

The camera matrices derived from the fundamental matrix described projections of the 2D feature points into a 3D scene, where each point became a ray. We computed the 3D coordinates of feature points by intersecting the two rays from corresponding feature points of left and right cameras, a process also termed triangulation. When two cameras were set up using the 45° viewing angle, the triangulation achieved the optimal precision because the camera’s optical axes were perpendicular. In contrast, the 90° viewing angle resulted in overlapping camera optical axes, which cannot be used to reliably determine intersections. Therefore, 3D reconstruction was only computed for the 45° videos.

The advantage of 90° videos was little perspective distortion because mouse M was walking on a straight line within an image plane perpendicular to the camera optical axes. We developed another script to compute postures from two camera angles of the 90° videos. To merge the feature points from the left and right cameras, we computed the average of corresponding points. Therefore, only 2D coordinates were available for the 90° videos. Similar to three-dimensionally reconstructed feature points, we transformed points of 90° videos to use the beam as the X-axis and mapped all coordinates to their physical scale.

### 2.8 Standard walk cycle extraction

We define the standard walk cycle (SWC) as a function of time (t) representing the speeds of all feature points with finite support, i.e., a cycle. The SWC is a recurring pattern to describe a mouse’s normal walking postures on the beam. To obtain the speed for each feature point, we used the finite difference method:


(1) V(t)=X(t)−X(t−Δt)

Where X(t) is a vector comprising all XYZ coordinates from all feature points $\left(x_{1},y_{1},z_{1},x_{2},y_{2},z_{2},\ldots \right)$ and Δt the time interval between two video frames (0.0333 s for 30-fps videos). Here, we use N to denote the set of coordinates and n for its component. For the 2D coordinates from the 90° videos, N contains 36 components; for the 3D coordinates from the 45° videos, N contains 54 components. We use the bold font to indicate a vector or vector-valued function of this layout, whereas the non-bold font with a subscript indicates its component.

When a mouse walks strictly following the SWC, its speed function is periodic:


(2) $$V_{SWC}\left(t\right)=V_{SWC}\left(t+\tau \right)$$

Where τ is the period of one cycle. A single cycle can be extracted using a window function W of width τ and left edge at $t_{0}=0$ :


(3) $$SWC\left(t\right)=V_{SWC}\left(t\right)\cdot W\left(t,t_{0},\tau \right)=V_{SWC}\left(t\right)\cdot rect\left(\frac{t-t_{0}}{\tau }-\frac{\tau }{2}\right)$$

Where rect() is a rectangular function. Ideally, the SWC is computed from V(t) by an optimization:


(4) $$\begin{array}{l} SWC\left(t\right),\;T=argmin_{T,\;SWC}\\ \left(\overset{\left|N\right|}{\underset{n=0}{\sum }}\overset{\left|T\right|}{\underset{i=0}{\sum }}SWC_{n}\left(\frac{t-t_{i}}{\tau_{i}}\right)-V_{n}\left(t\right)\cdot W{\left(t,t_{i},\tau_{i}\right)}_{2}\right) \end{array}$$

Where T is a set of all $\tau_{i}$ , the length of each cycle, and $t_{i}$ , the start time of each cycle. They obey the following relationship:


(5) $$t_{i}=\overset{i-1}{\underset{j=0}{\sum }}\tau_{j}$$

In Eq. 4, we want to compute an SWC that minimizes its speed difference, measured by the L2 norm, to an input V(t) when each cycle is duplicated from SWC under shift (of start time at $t_{i}$ ) and scale (of period $\tau_{i}$ ). Since both T and SWC need to be determined for the optimization, a direct evaluation of Eq. 4 is difficult.

**Figure 5:**
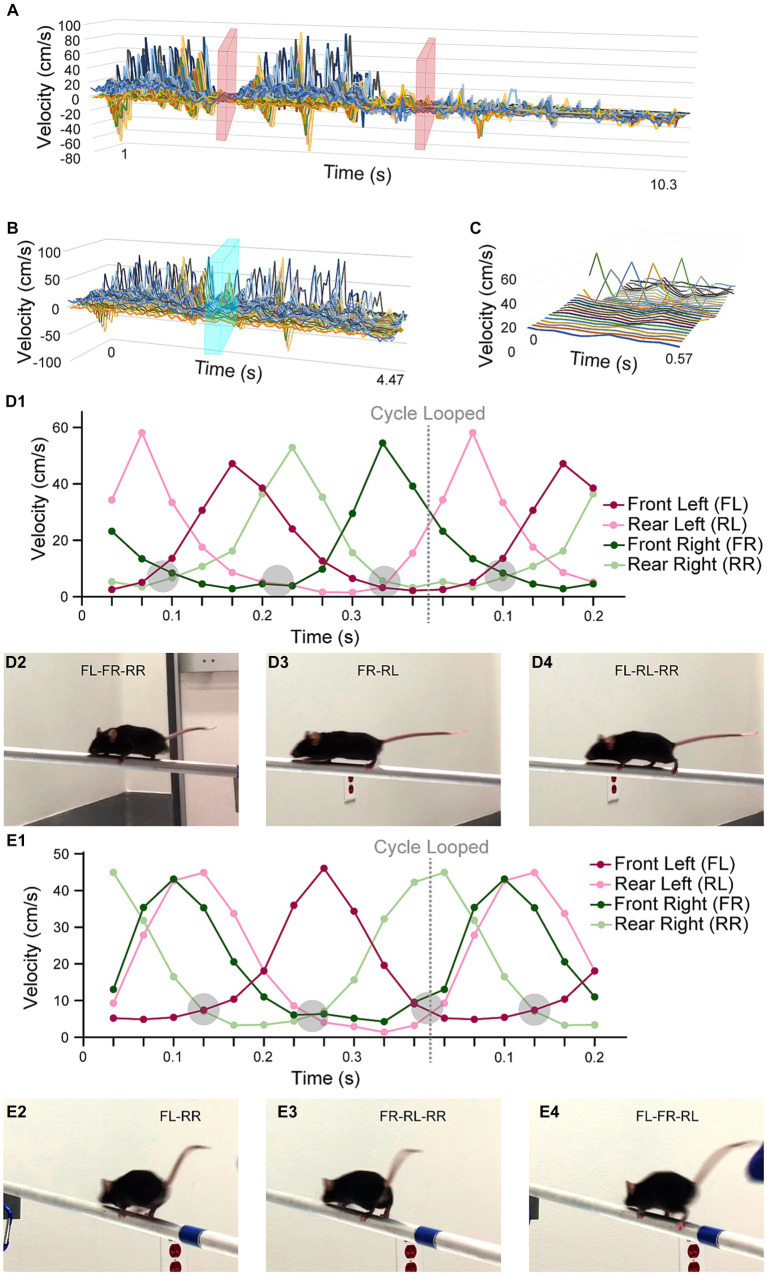
Standard walk cycle extraction. All horizontal axes are time (unit = m/s), which were captured at a 30-fps rate. **(A)** A graph showing the speed values over time for all XYZ speed components and all feature points. Time points when the mouse was not moving on the beam were cropped in the video analysis portion (pink planes). **(B)** The optimization process for computing the SWC was initialized by a manually selected cycle, illustrated by the cyan plane. **(C)** The SWC was plotted with all its XYZ speed components and feature points in a 3D graph. The most prominent peaks were from the movements of the feet in the X direction. **(D1)** A graph only showing the X speed of the four feet of mouse M. We detected a consistent pattern of supportive legs, at points where multiple curves crossed at near-zero speed (encircled grey regions). **(D2-4)** The still frames when the mouse was supported by 2 or 3 legs. It was easily observed from the still frames because the 30-fps video capture rate made moving legs blurry. **(E1)** A graph showing the X speed of the feet of mouse F. The same pattern of supportive legs was observed. However, mouse F tended to move two legs (FR and RL) at the same time. The pattern of supportive legs was also mirrored to that of mouse M. **(E2-4)** The still frames when mouse F was supported by 2 or 3 legs.

We considered a SWC of the same temporal resolution as the input videos to be sufficient for subsequent analysis. Therefore, we developed a heuristic method to compute a discrete SWC in steps. First, we culled the input V(t) to remove undesired data, which were time points when a mouse stopped briefly on the beam or before entering the destination box (Figure 5A). Second, we examined the culled V(t) and manually selected an interval as the initial condition for SWC. In Figure 5B, the most prominent peaks of V(t) were from the foot movements. Empirically, we used prominent peaks from feet movements to identify a repeating pattern and chose the initial condition to best match the pattern. Third, the initial condition $SWC_{0}$ was used as a template to search for matching cycles from V(t) . This was achieved using a simplified optimization from Eq. 4:


(6) $$\begin{array}{l} T=\arg\max_{T}\\ \left(\overset{\left|N\right|}{\underset{n=0}{\sum }}\overset{\left|T\right|}{\underset{i=0}{\sum }}corr\left(SWC_{0,n}\left(\frac{t-t_{i}}{\tau_{i}}\right),V_{n}\left(t\right)\cdot W\left(t,t_{i},\tau_{i}\right)\right)\right) \end{array}$$

Here, we computed T by maximizing the correlation between V(t) and a speed function generated by repeating $SWC_{0}$ under shift and scale. In other words, we used the $SWC_{0}$ as a template image and performed image registration to obtain subdomains T in V(t) , so that each subdomain $\left(t_{i},\tau_{i}\right)$ of V(t) best matched $SWC_{0}$ . Since we were computing using discrete-time series V(t) , we fixed the search space using a series of periods ranging between half to double the length of $SWC_{0}$ . A maximum correlation sum was obtained by a full search on the finite grid of all candidate T s. Once T was determined, the SWC was computed by a weighted average:


(7) $$SWC\left(t\right)=\frac{\sum_{i=0}^{\left|T\right|}\left(C_{\max,i}\cdot V\left(t\right)\cdot W\left(t,t_{i},\tau_{i}\right)\right)}{\sum_{i=0}^{\left|T\right|}C_{\max,i}}$$

Where $C_{\max}$ is the set of maximum correlation values corresponding to the T obtained from evaluating Eq. 6. In theory, one SWC was uniquely computed for one mouse from all its videos. In practice, we accommodated the progressive refinement of the SWC when videos of the same mouse were analyzed sequentially. We designed a file format to save the SWC along with the weight $\overset{\left|T\right|}{\underset{i=0}{\sum }}C_{\max,i}$ . When a new video was added to an existing analysis, we reevaluated Eq. 6, replacing $SWC_{0}$ with the previous SWC(t) . Then, we computed the weighted average in Eq. 7 using both the new weights and the weight from the previous SWC(t) .

### 2.9 Synthetic walk generation

We obtained $V_{SWC}\left(t\right)$ by repeating SWC indefinitely over time. Under an initial condition $X_{0}$ , the synthetic posture of a mouse under SWC is:


(8) $$X_{SWC}\left(t\right)=X_{0}+\overset{t}{\underset{0}{\int }}V_{SWC}\left(t\right)dt$$

We maintained the time interval Δt from the input videos captured at 30 fps. Therefore, we numerically evaluated Eq. 8 using the Forward Euler method:


(9) $$\{\begin{array}{c} X_{SWC}\left(0\right)=X_{0}\\ X_{SWC}\left(t+1\right)=X_{SWC}\left(t\right)+V_{SWC}\left(t\right) \end{array}$$

However, since SWC was computed by averaging walk cycles from V(t) , the condition for the synthetic walk returning to the posture of $X_{0}$ after one period τ was usually not satisfied:


(10) $$\overset{\tau }{\underset{0}{\int }}V_{SWC,n}\left(t\right)dt\equiv V,\forall n\in N$$

Where V is a constant 3D vector representing the overall movement in one SWC. To make meaningful synthetic walk animations, we normalized $V_{SWC}\left(t\right)$ to enforce Eq. 10. Considering the movement of a mouse was constrained by the beam, we computed V by finding the maximum displacement in the X (beam) direction and setting YZ to 0:


(11) $$V=\underset{n_{x}}{\max}\left(\overset{\tau }{\underset{0}{\int }}V_{SWC,n_{x}}\left(t\right)dt,0,0\right)$$

The synthetic walk generation was implemented using a FluoRender script. The initial condition $X_{0}$ was drawn using the ruler tools in FluoRender. After running the script, the results were viewed three-dimensionally using the movie playback functions in FluoRender. The animation for the perspective view in Videos 1&2 was generated by key-frame functions in FluoRender to track mouse body displacement with the camera.

### 2.10 Event abstraction

We compared the SWC against the input V(t) from each video to detect when and how much the walk deviated from the SWC. The result was a quantitative description of the events during a mouse’s walk. First, using the set of periods T from Eq. 6, we computed a matrix of the correlation values for each period ( $\tau_{i}$ ) and feature point axis (n). The values of the correlation matrix were normalized using the autocorrelation of SWC as a reference. Typically, a value of 0 indicates no movement and 1 indicates a match with the SWC; values higher than 1 indicate movement at higher speed than the SWC; negative values indicate movements away from the directions of the SWC. Although the correlation values provided a detailed quantification of the walk, the amount of data from multiple feature points and XYZ directions made it difficult for users to understand. Some anomalies, such as slips, jumps, and hesitations, were observed in the videos. But their relationships to the correlation values were not instantly obvious to users. Therefore, further abstraction for easy interpretation was needed.

We derived the variance of correlation (VoC) from the correlation values in two steps. First, we grouped the feature points according to the body parts of a mouse: head, body, tail, and feet. Then, we computed the second central moment (variance) for the correlation values per group and per period ( $\tau_{i}$ ). We plotted VoC against time by maintaining its value within each period ( $\tau_{i}$ ). The computing of VoC produced a simpler quantitative representation for mouse movements: VoC is non-negative; a low value near 0 indicates little deviation from the SWC or no movement; VoC increases when there is a significant deviation of movements from the SWC.

### 2.11 Analysis and statistics

Data are shown as mean +/− SEM and statistical analysis was performed using one-way ANOVA to compare all groups within each category, including mouse strain, age, body mass, and sex. Within each category, two-tailed t-tests were used to complete pairwise comparisons, separately. Statistical significance is denoted as *p*-values between 0.01 and 0.05 (*), 0.001–0.01 (**), 0.001–0.0001 (***), <0.0001 (****), while any value of *p* > 0.05 is not significant (ns).

## 3 Results

We designed a workflow for analyzing the postures of transgenic mice walking along the balance beam for 60 cm (Figure 1). All computation and visualization tasks in this workflow were accomplished using an integrated system with an intuitive user interface, FluoRender. We developed new tools and scripts based on the FluoRender architecture, which were included within the latest release (v2.29.3) and can be downloaded from github.com.^1^ We performed the posture analysis by first opening a video file in FluoRender. Then, we used FluoRender’s ruler tools to create feature points as training examples. We executed the script “video_train” from the “Script” subpanel in the “Record/Export” panel to train a DLC model from the examples. To apply the trained model, we executed another script “video_analysis,” which generated the tracked feature points for each video frame. We used the script “ruler_speed” to generate the speed information on the tracked points; we used the script “stereo_reconstruct” to reconstruct a 3D tracking from a pair of 45° videos; we used the script “gen_walk” to generate a 3D synthetic walk from a SWC. More details on FluoRender scripts including the specific scripts for posture analysis can be found in Chapter 13, Automated Data Analysis with Scripts, of the online manual, which can be accessed from the main menu of FluoRender.

We examined transgenic and wild type mice traversing a balance beam by evaluating their dynamic gait, and motor coordination by tracking a single point on the mouse. We initially evaluated performance of female and male adult mice, with different body masses by tracking a single point on the mouse. Gad2-Cre::GCaMP5G-tdTomato (Gad2-G5-tdT), Gad2-Cre, PC::GCaMP5G-tdTomato (G5-tdT), and parental strain, C57BL/6 J in adults. We observed the Gad2-G5-tdT mice with small, but significant deficits in velocity based on age, strain, and sex (Figures 2C–E; Table 1).

**Table 1:** Statistical analysis of balance beam assessments.

Figure	Parameter and comparison	Statistical test	*p*-value	Significance
2B	Mean velocity by age	One-way ANOVA	0.0006	***
2C	Mean velocity by age category	Two-tailed *t*-test	0.0453	*
2D	Mean velocity by strain	Two-tailed *t*-test	0.0274	*
2E	Mean velocity by sex	One-way ANOVA	4.671e-13	****
2F	Mean # of stops by sex	One-way ANOVA	0.1361	ns
2F	Mean # of stops (WT Males v. WT Females)	Two-tailed *t*-test	0.0452	*

ns *p* > 0.05, *p ≤ 0.05, **p ≤ 0.01, ***p ≤ 0.001, ****p ≤ 0.0001.

Next, we tracked and analyzed a total of 20 videos in 10 left–right pairs per mouse using a multidimensional function. We used FluoRender to generate tracking examples from select still frames. We developed a FluoRender script to feed these examples to the DeepLabCut (DLC) library and perform model training. Videos of different viewing angles, i.e., left 45°, right 45°, left 90°, and right 90°, were grouped and separate models were trained for each group. We developed a second FluoRender script to apply the models to all videos, so that every frame was labeled with feature points representing mouse postures. We used FluoRender to examine and curate the crude tracking results (Figure 3). We frame matched videos from each left–right pair and then combined their feature point coordinates. Therefore, one time sequence of postures was produced for each video pair. For the 90° video pairs, we computed the feature points’ 2D coordinates as the mean values of corresponding points. For the 45° video pairs, we reconstructed their feature points’ 3D coordinates by first computing the fundamental matrix and then triangulating from corresponding points (Figure 4). We also traced the beam in FluoRender to map points in the 2D/3D scene to their real-world scale using the measured length of the beam.

We computed the speed (a 2D vector for 90° video pairs and a 3D vector for 45° video pairs) per point per video frame using the finite difference method. We culled the speed data to only keep the frames when the mouse was walking on the beam (Figure 5A). We extracted walk cycles from the speed data using a heuristic optimization algorithm (Figures 5B,C; Eq. 6). The correlation of each walk cycle to the SWC was used as the weight. One SWC was computed for each mouse. Their graphs are in Supplementary Figures S1, S2. The SWC was used to describe how a mouse walked typically on the beam. Our examination of the SWCs was focused on the foot movements. We found both similarities and diverging characteristics between the two mice in our experiments (mouse M for the male mouse and mouse F for the female mouse). First, the temporal pattern of the number of supportive legs (i.e., legs on beam supporting body in one frame) was consistent. In Figures 5D1,E1, we observed that their four legs were moving in well-defined order. The body was supported by two or three legs in a repeating 2–3-3 pattern. However, the leg-dominance differed between mouse M and F, as the pattern was mirrored laterally to the moving direction: (FR-RL)-(FL-RL-RR)-(FL-FR-RR) for mouse M vs. (FL-RR)-(FR-RL-RR)-(FL-FR-RL) for mouse F. This alternating pattern also indicated that the time intervals between leg movements were unevenly distributed. We detected two closely grouped peaks in the SWC graphs in Figures 5D1,E1: FL and RR for mouse M and FR and RL for mouse F. For mouse F this grouping of FR and RL was more obvious, which exhibited as these legs moved together consistently the majority of the time.

**Figure 6:**
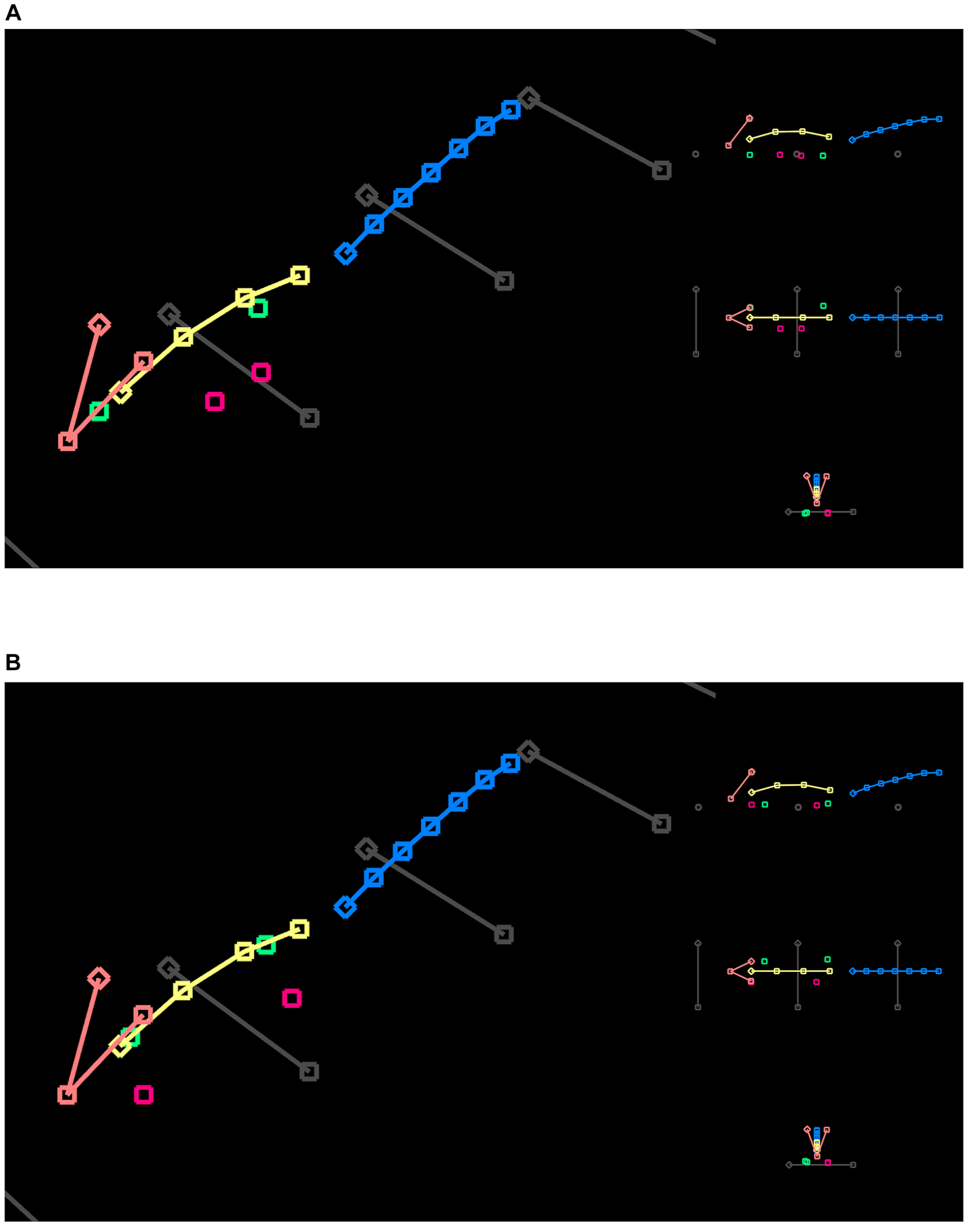
**(A)** A single frame from the synthetic walk reconstructed from the SWC for mouse M. The SWC is 2D because of the camera angles. Therefore, there is no side movement from the orthographic views (Supplementary Video S1). **(B)** A single frame from the synthetic walk reconstructed from the SWC for mouse F. The SWC is 3D, representing the averaged postures from all videos that recorded mouse F traversing the beam (Supplementary Video S2).

We generated synthetic walk animations from the SWC for each mouse (Figure 6). A SWC was normalized to remove inconsistent movements among the feature points. We drew a 3D initial condition in FluoRender and computed its displacements over time as an integral using the forward Euler method. Supplementary Video S1 shows the synthetic walk of mouse M, viewed from different angles. Supplementary Video S2 shows the synthetic walk of mouse F. Since only 2D feature points were available for mouse M, there was no lateral movement in its synthetic walk, despite its initial condition being drawn in 3D. Supplementary Video S2 shows more realistic walk cycles when lateral movements were included from 3D reconstructed coordinates. The synthetic walk animations provided a means to visualize the SWC in an intuitive manner.

**Figure 7:**
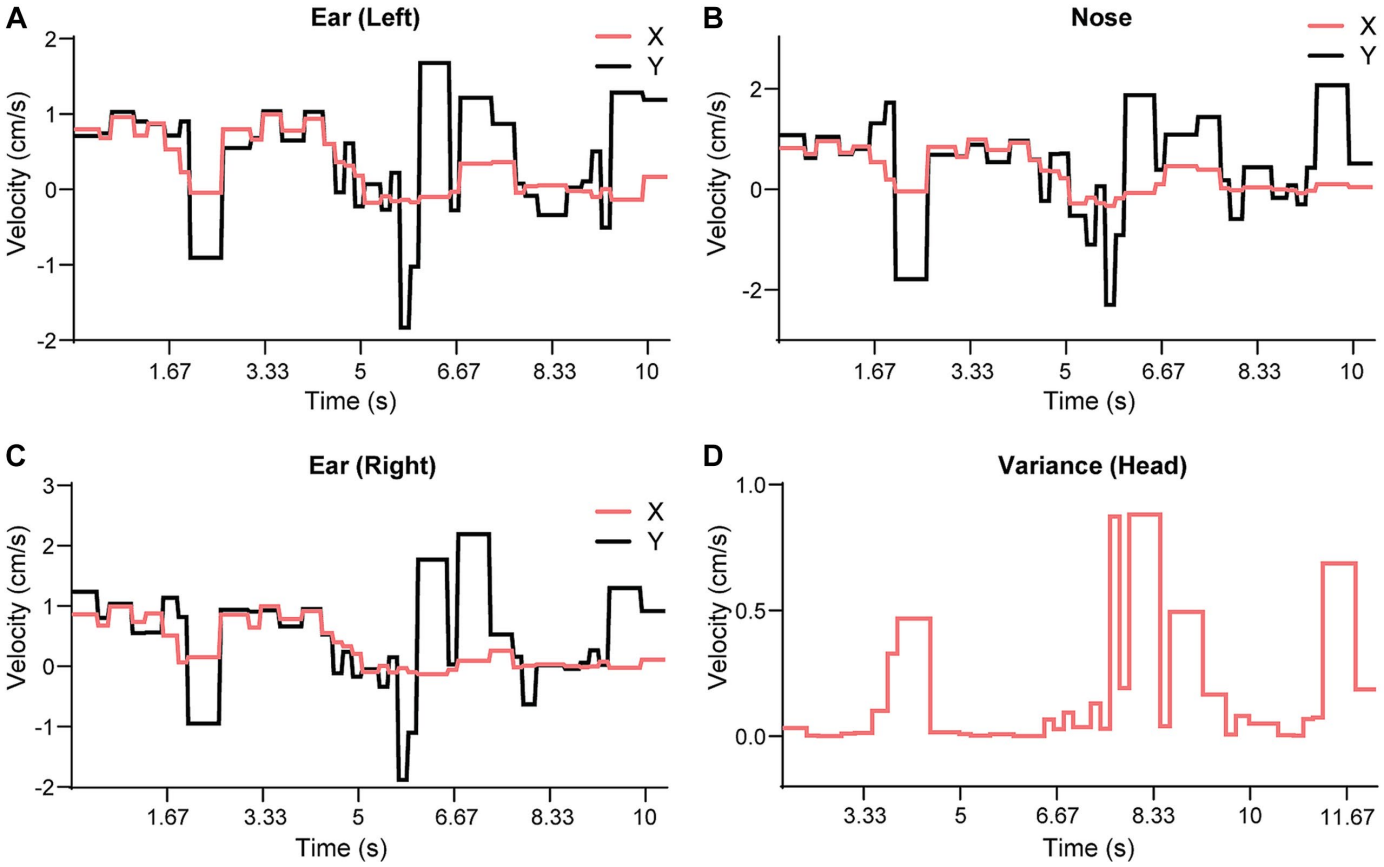
Comparison of SWC against the speed data. All horizontal axes are time counted as video frames. **(A)** video frame is one thirtieth of a second. **(A–C)** The results show three feature points on the mouse head from Supplementary Video S3. We computed the correlation values for each speed component (X and Y for the 90° configuration) and each feature point. The correlation values were normalized using a scale where 0 indicates no movement and 1 indicates a match to the SWC within one cycle. **(D)** We computed a variance measure (VoC) from all speed components and feature points. This measure is easier to read because it is non-negative and high values indicate large deviations from either the SWC or static state.

**Figure 8:**
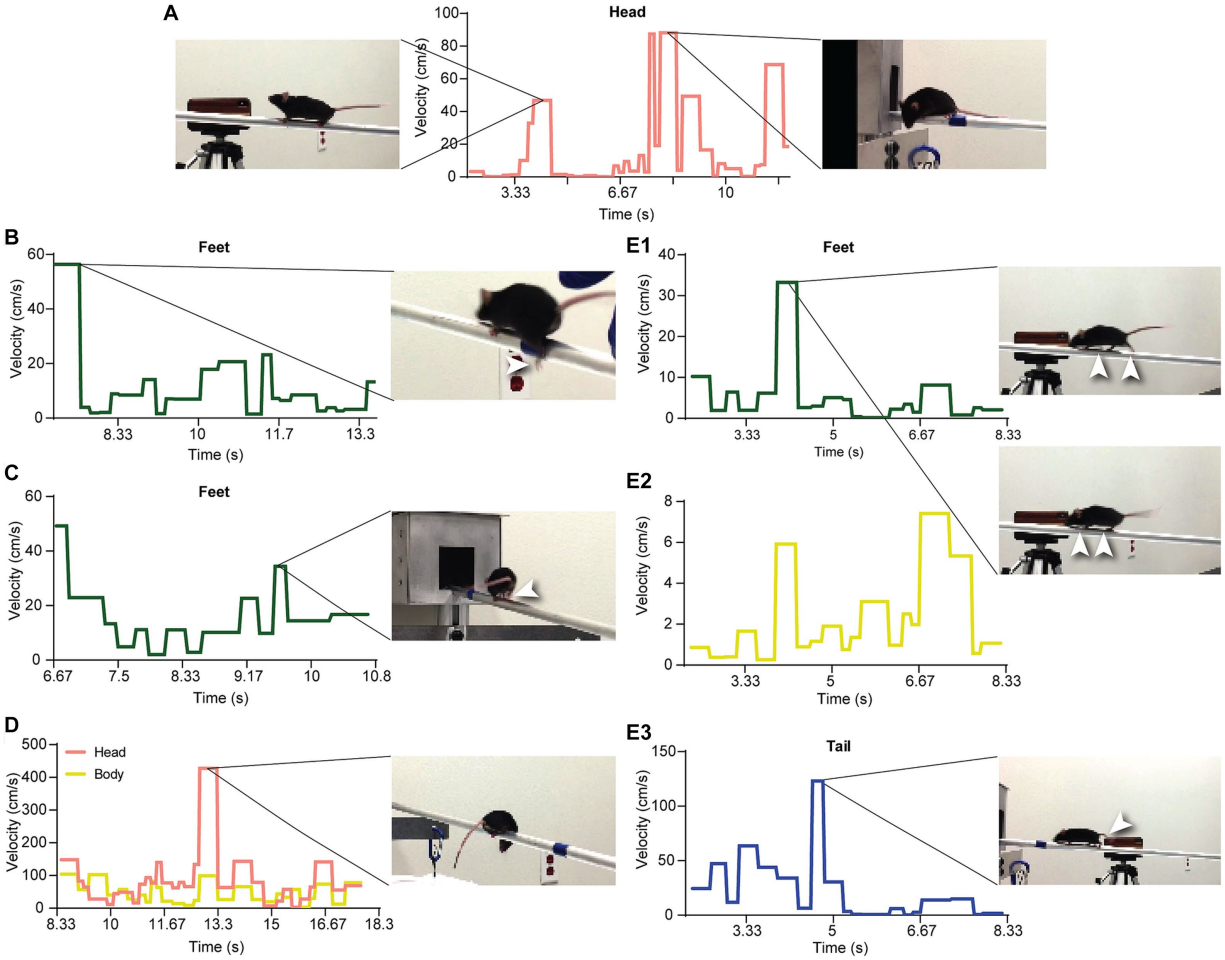
Detection of events and anomalies using VoC. **(A)** The VoC of the head. The first peak is from when mouse M stopped and raised its head. The flat lines surrounding the first peak are from walking postures very close to the SWC. The peaks of the later half are from head movements before entering the box. **(B)** A peak from the VoC of the feet is from a slip when mouse F just started moving. **(C)** Mouse F jumped (both rear legs moving at the same time) when approaching the box. **(D)** Mouse F looked down below the beam, resulting in a high peak of the head VoC. **(E1,2)** The supportive leg pattern differed from the SWC. We observed brief hesitation of the mouse’s movements. **(E3)** The compensational movements of the tail, delayed by about two cycles, were observed after the abnormal movements of the legs.

We used the SWC to quantitatively evaluate the movement and behavior of a mouse on the beam. First, we extracted individual walk cycles from the posture data for each video by an algorithm akin to image registration, where the SWC was used as a template image. For each walk cycle, we computed its correlation to the SWC, which measured the degree it deviated from the SWC. Figures 7A–C show the correlations for the XY speed of the three feature points of the head of mouse M. Here, we normalized the correlation values so that 0 indicated no movement and 1 for an exact match to the SWC. The correlation to the SWC provided detailed information on movement for each feature point but was not intuitive to interpret. We further organized the feature points into groups of head, body, tail, and feet. A single variance value was computed for all correlation values of feature points at each time point in each group. Figure 7D shows the variance of correlation (VoC) for the head group. The VoC provided a concise description of movement. Figure 8A shows the VoC of the head movement of mouse M, which walked halfway on the beam and stopped. The mouse raised his head and looked. Then, the mouse started walking and stopped again before the box at the other end of the beam. Another example is shown in Figure 8D for mouse F. The peaks of the VoC curve matched activities deviated from the typical walk posture. Supplementary Video S3 is an example of a composition including both the original video and VoC graphs, where we used red vertical lines to indicate current time for easy comparison. The VoC graphs revealed the characteristics of how a mouse walked. For mouse M, there existed a strong anti-correlation between its head and tail movements. Non-SWC movements of its head never overlapped with those of its tail, suggesting a certain compensative role of the tail. However, this relationship between head and tail was not always obeyed by mouse F. We examined the consistency of footsteps, which was revealed by the VoC graphs of the foot group. We observed that mouse F walked with less consistency as the VoC values fluctuated at high levels. Notably, mouse F’s walk cycle deviated more significantly from the SWC when it just started walking. Mouse F often had a foot slipping off the beam (Figure 8B). Therefore, we also used the VoC graphs to detect anomalies in walk cycles, such as slipping, jumping, and phase shifting. Figure 8 shows typical anomalies that we found using the VoC graphs in the videos. We were able to draw a patten on temporal relationships among different groups. For example, the radical tail movements exhibited as a VoC peak in Figure 8E3 was likely a compensation to a brief stall/hesitation just 2 cycles earlier (Figures 8E1,2).

## 4 Discussion

### 4.1 Balance beam test

The balance beam test provides quantitative information on mouse movements at three levels of detail. First, the most simple and common analysis is to record the time to traverse a known distance on the beam, and calculate the average velocity. Many events during the traverse, such as brief stops, cannot be effectively recorded using only the average velocity. Second, video recordings of the mouse are leveraged to track the traverse as a single point moving over multiple video frames. Third, a multidimensional function is generated by tracking the pose of the mouse, as described in this study. Our workflow provides more detailed information on subtle movements of the mouse in each frame. The choice of the data analysis depends on the research goal and cost of effort. In this study, speed data were gathered and analyzed from 50 videos to establish a broad scope of the performance (Figure 2). Then, we focused on two mice (mouse M and F) and performed studies on their posture (Figure 8 and Supplementary Video S3). More effort was demanded to fully analyze all 50 videos. Therefore, we focused on 20 videos to capture the details presented here.

### 4.2 Camera position

Comparing the 45° and 90° viewing angles for video capture, we recommend the 90° setup for its simplicity and effectiveness. Three-dimensional reconstructed postures are optional because the movement of the mouse is constrained by the beam. When the optical axis of the camera is not at a right angle to the beam (i.e., 90°), perspective correction is required for correct speed evaluation. The information on Z movement allowed us to generate errorless synthetic walk animations. However, we only used XY speed values to compute the VoC for both mice male (M) and female (F). It allowed us to compare the results from both mice using identical calculations. A significant disadvantage of the 45° setup is its decreased quality when the mouse moves further away from the camera. Fewer pixels are utilized when the image of the mouse becomes smaller (i.e., further away) because of foreshortening. The front of the mouse is more easily occluded, making accurate tracking difficult. For the 90° setup, the cameras are near the center of the beam and potentially distractive to the mouse during walk. This may be more problematic when larger cameras are used. Cameras with shift (perspective control) lenses can be placed near the start end of the beam while keeping the optical axes perpendicular to the beam. A more cost-effective method we recommend is camera camouflage that blends it into the environment.

### 4.3 Video resolution and frame rate

We regard standard HD videos (1,280 by 720 pixels at 30 fps) from contemporary smartphones sufficient. Image clarity for accurate tracking is achieved by the combination of image sensor and optics more than the increase of resolution setting. Simply setting a high video resolution only results in larger files and prolonged processing time for model training. Similarly, increasing the frame rate by camera setting or dedicated high-speed imaging equipment also makes data processing and analysis costly. An advantage of using a relatively low 30-fps rate is easy distinguishing between motion and inactivity from individual video frames, which might seem counterintuitive initially. Figures 5, 8 contain examples of motion blur from quick movements. We leveraged the motion blur as an indicator to identify the supportive legs for a mouse during its walk. Examining a frame from a high-speed camera becomes more laborious when there are more frames to analyze, as it will require the user to check multiple adjacent frames to identify a leg in motion. For manual tracking and curation, when the placement of a feature point became uncertain because of motion blur, we placed the point at the center of the blurred region.

### 4.4 DeepLabCut^™^ (DLC)

The bottleneck of deep learning in practice is model training. In our workflow, we provided training examples from manual work and generated models using available computing resources. Based on the timing of manual curation, we estimate that the net performance of a full manual tracking process is at least on par with our semi-automatic workflow. As the cost of human effort becomes prohibitive when the number of input videos increases, a workflow mixing manual work and deep learning is needed. We found it unfeasible to train a perfect model for error-free tracking using DLC. For example, we used every curated frame in a video to refine a DLC model, which was then applied to track the same input frames. Although there was a significant improvement to the original model, the tracking results from the refined model did not match the curated frames. Since errors are unavoidable from a machine-learning generated result, a hybrid workflow involving manual examination and correction is needed to ensure the quality of data analysis. When multiple videos are present for tracking, we recommend an iterative refinement of the model: (i) choose a video with the least complex movements and generate tracking examples with approximately 10% of total frames; (ii) train a model with the examples and apply it to the video; (iii) curate all frames of the first video; (iv) use the curated frames to refine the model; (v) choose a second video and track it with the refined model; (vi) examine and curate the second video; (vii) skip model refinement if errors diminish; (viii) repeat (v-vii) for the remaining videos and only refine the model if common errors in Figures 3A–G increase whereas special cases like Figure 3H do not need refinement.

### 4.5 Manual curation

Refinement of a DLC model is limited by both training examples and DLC model capacity, which makes manual curation necessary for tracking quality. We demonstrated the efficiency of manual tracking is significantly improved by designing a workflow with accompanying tools. The general goal of designing our tools is to minimize the travel distance of a computer mouse for curation. Changing video frames is a commonly used function in manual curation, which is operated by mouse clicks on a timeline control within the user interface. However, this operation increases mouse travel distance and diverts user attention from the feature points. Frequent switches the focal point between the tracking results and controls within the user interface quickly cause fatigue. Therefore, keyboard shortcuts in FluoRender are recommended for changing video frames. Switching among different editing tools is another cause of fatigue. We repurposed and multipurposed existing FluoRender ruler editing tools based on the context of use to make a single tool intuitive for different curation tasks without user-enacted tool switching. For example, the move tool is normally operated by dragging a ruler point, but behaves like a magnet tool when operated by single-clicks; dragging without selecting a point temporarily enables view port manipulations, such as panning and zooming. Furthermore, we found that the operations for curation were streamlined at high efficiency when only one feature point was edited through all frames. Therefore, we provided ruler masking in FluoRender so that only the selected rulers are shown or modified within a focused scope. Nevertheless, manual curation requires considerable time and labor when the number of video frames increases. For projects with time constraints, some errors can be tolerated to save time for correcting high impact errors. The determination of the impact of an error on the quality of analysis is project dependent. In our work, a common but high impact error is the switching of left and right points in Figures 3B,E, which results in an erroneous speed spike. It also requires more manual examination to detect and correct. Inaccurate placement of points, for example, on feet because of motion blur, usually have less impact and can waive curation for high throughput processing.

### 4.6 Standard walk cycle (SWC) and analysis

The SWC is an average of real walk cycles, which differ from the SWC with varying amplitudes and phases. The feature points in a real walk cycle can phase shift altogether, e.g., when a mouse stops briefly, or independently as in a jump (Figure 8C) or an abnormal supportive leg pattern (Figure 8E). The internal phase shifts influence the shape of the SWC. In Figure 5, the foot speed curve of mouse M is more pointed at peaks than mouse F. This is because internal phase shift was less frequent for mouse M, an indicator of its walk being consistent. Expanding the solution space by allowing more degrees of freedom of $SWC_{0}$ when evaluating Eq. 6 can compensate for internal phase shift and improve the temporal resolution of the SWC. However, the computing cost may also increase significantly. A fixed $SWC_{0}$ allowed us to accomplish a full search of the solution space at interactive speed, i.e., the result was computed before playing back through a video. Another simplification we made in Eq. 6 was measuring distance between two functions by correlation instead of the L2 norm. In addition to simpler calculation, computing correlations has the benefit that the resulting values are directly used as weights for averaging in Eq. 7. Furthermore, we used the same algorithm and code to compute the correlations to analyze the deviations of walk cycles from the SWC.

## 5 Conclusion

The balance beam is an established method for studying rodent vestibular function with unconstrained head movements. In this study, we presented an integrated workflow for analyzing the postures of a mouse traversing the balance beam. Our workflow combined DLC and FluoRender’s ruler tools, including the “magnet tool,” “move tool,” and “redraw tool,” for tracking feature points accurately. Although DLC offers immense benefits in markerless pose estimation, fully automatic and accurate tracking using AI cannot be achieved at this time without manual curation. Our workflow provides manual curation in FluoRender as an alternative to the built-in tool in DLC to improve the usability and efficiency. The integration of analysis functions in FluoRender scripts allowed us to further process the speed information obtained from pose estimation and generate quantitative measurements on movements and behaviors. We first extracted a standard walk cycle (SWC) from the repetitive patterns in mouse movements. Then, we used the SWC to quantitatively evaluate the degree of deviation in mouse movements. We further derived the variance of correlation (VoC) as a measurement of anomalies in a group of connected feature points. We were able to detect events such as stops, slips, and foot phase shifts using only VoC values, which reduced the amount of time needed to examine the video files. In future studies, our workflow can be integrated with eye tracking (20), and two-photon voltage or calcium imaging with cranial windows. Combining these tools could offer a multimodal technique to study spatiotemporal neuronal signaling *in vivo* while testing the vestibular system.

## Data availability statement

The datasets presented in this study can be found in online repositories. The names of the repository/repositories and accession number(s) can be found in the article/Supplementary material.

## Ethics statement

The animal study was approved by University of Utah Institutional Animal Care & Use Committee (IACUC). The study was conducted in accordance with the local legislation and institutional requirements.

## Author contributions

YW: Conceptualization, Data curation, Methodology, Software, Writing – original draft, Writing – review & editing. ME: Formal analysis, Writing – review & editing. CK: Data curation, Investigation, Validation, Writing – review & editing. JS: Investigation, Validation, Writing – review & editing. HH: Conceptualization, Data curation, Formal analysis, Funding acquisition, Investigation, Methodology, Project administration, Resources, Supervision, Validation, Visualization, Writing – review & editing, Writing – original draft.

## Footnotes

^1^https://github.com/SCIInstitute/fluorender/releases/tag/v2.29.3

## Supplementary material

SUPPLEMENTARY FIGURE S1 The SWC of mouse M. The SWC contains 18 feature points in 4 groups of head, body, tail, and feet, which are organized in 4 rows. The first column is for speed values in the X (beam) direction, the second column for Y, and the third XY combined. All horizontal axes are video frames and one frame is a thirtieth of a second. All vertical axes are time (seconds).

SUPPLEMENTARY FIGURE S2 The SWC of mouse F. The SWC contains 18 feature points in 4 groups of head, body, tail, and feet, which are organized in 4 rows. The first column is for speed values in the X (beam) direction, the second for Y, the third for Z, and the fourth for XYZ combined. All horizontal axes are video frames and one frame is a thirtieth of a second. All vertical axes are time (seconds).

SUPPLEMENTARY Video S1 The full synthetic walk video reconstructed from the SWC for mouse M. The SWC is 2D only because of the camera angles did not support 3D reconstruction. There is no side movement, which can be observed from the orthographic views (Fig. 6A).

SUPPLEMENTARY Video S2 The full synthetic walk video reconstructed from the SWC for mouse F. The SWC is 3D, representing the averaged postures from all videos that recorded mouse F traversing the beam (Fig. 6B).

SUPPLEMENTARY Video S3 A composition of the original video (top left), a combined VoC graph containing all 4 groups of feature points (bottom left), and VoC graphs for individual groups (right column). They show relationships between the VoC values over time and events (deviations from SWC) in the video. This is an example of an original video (Mouse M).
