# Supplementary figures and images for: An integrated workflow for 2D and 3D posture analysis during vestibular system testing in mice
Source: Front Neurol. 2023 Dec 1;14:1281790. doi: 10.3389/fneur.2023.1281790 (PMC10722188; doi:10.3389/fneur.2023.1281790)

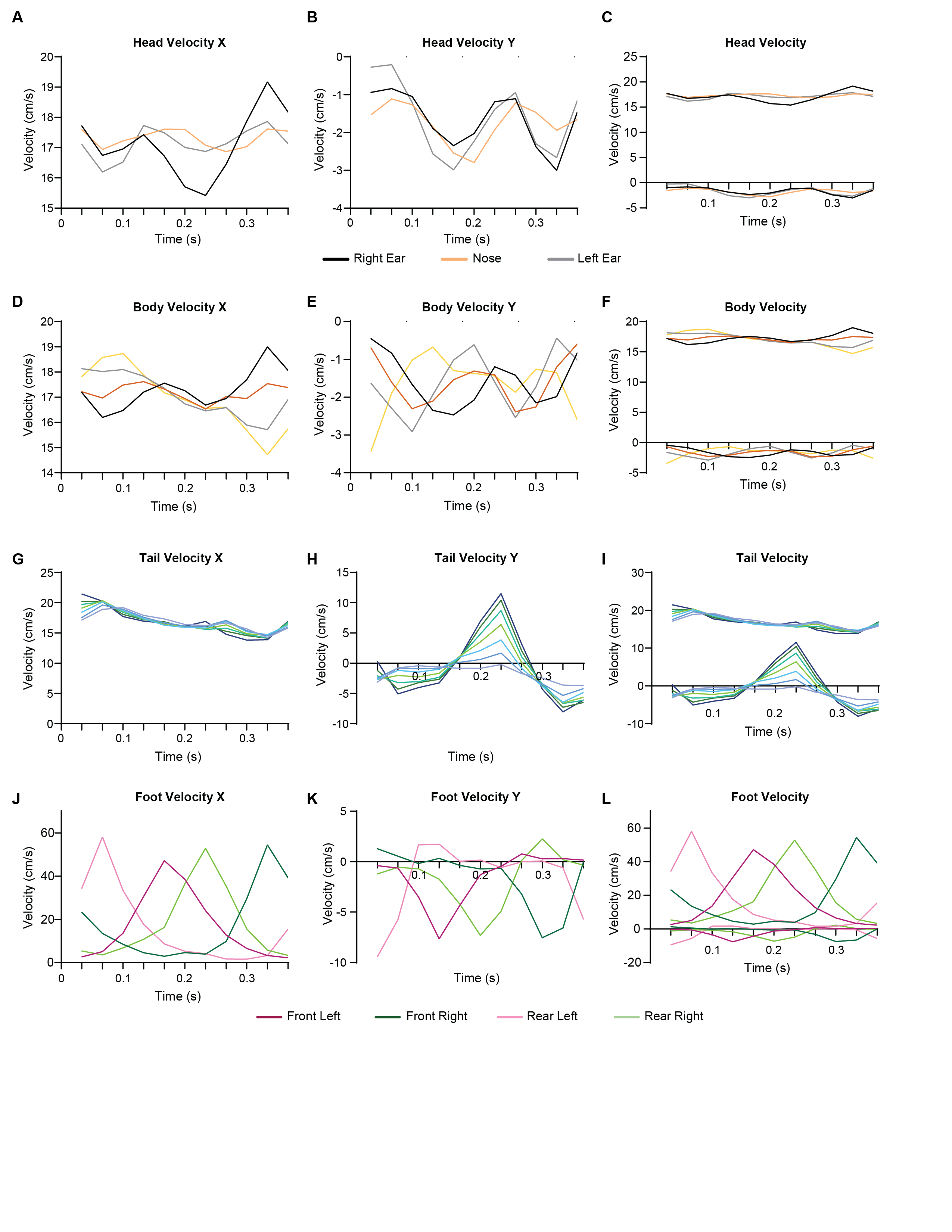

Supplement: SUPPLEMENTARY FIGURE S1 — The SWC of mouse M. The SWC contains 18 feature points in 4 groups of head, body, tail, and feet, which are organized in 4 rows. The first column is for speed values in the X (beam) direction, the second column for Y, and the third XY combined. All horizontal axes are video frames and one frame is a thirtieth of a second. All vertical axes are time (seconds). [file Image_1.TIF]

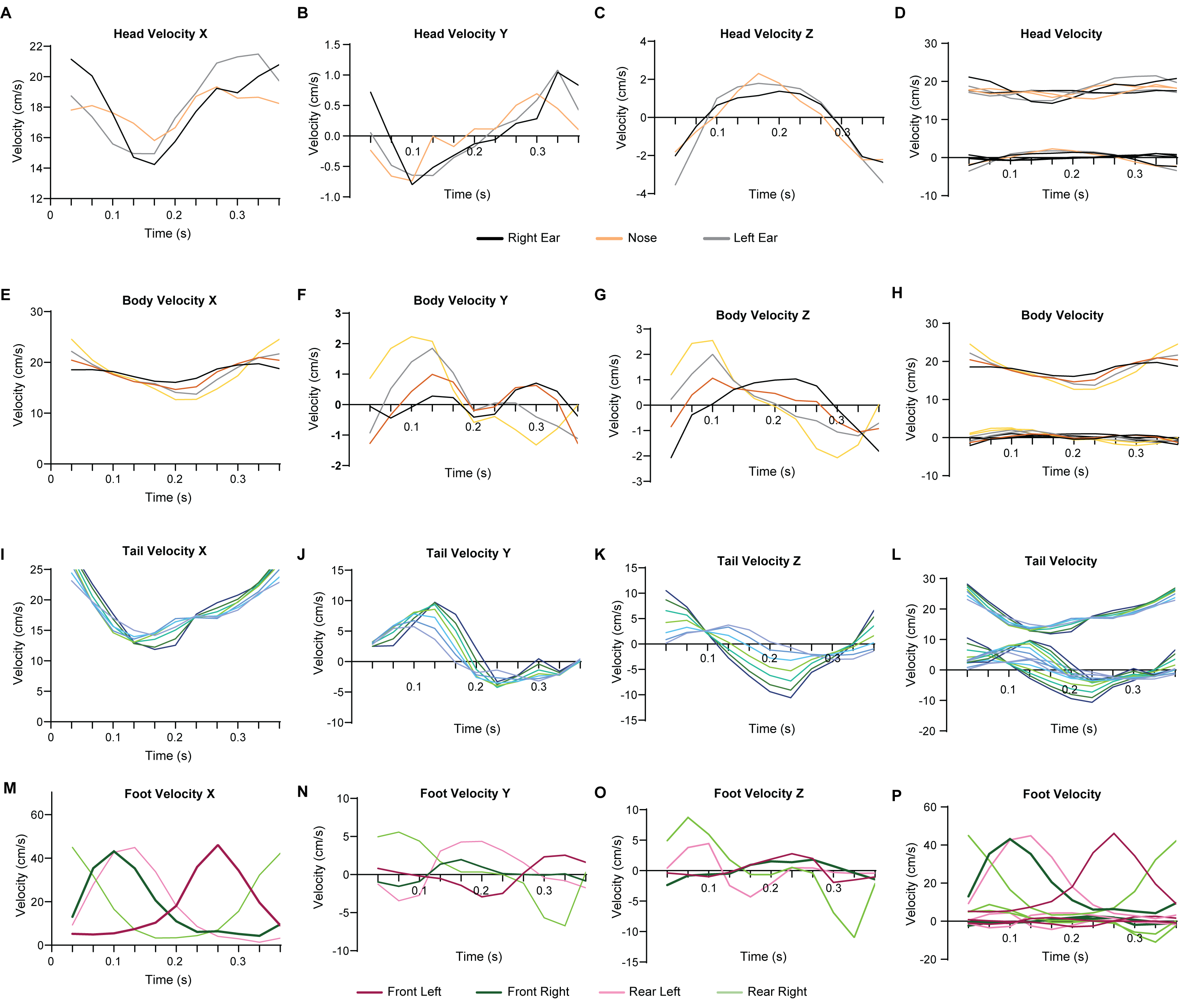

Supplement: SUPPLEMENTARY FIGURE S2 — The SWC of mouse F. The SWC contains 18 feature points in 4 groups of head, body, tail, and feet, which are organized in 4 rows. The first column is for speed values in the X (beam) direction, the second for Y, the third for Z, and the fourth for XYZ combined. All horizontal axes are video frames and one frame is a thirtieth of a second. All vertical axes are time (seconds). [file Image_2.TIF]
